# Extracorporeal Septoplasty for Severe Nasal Septal Deviation: A Systematic Review

**DOI:** 10.1002/lio2.70280

**Published:** 2025-10-10

**Authors:** Matteo Lazzeroni, Jèrôme René Lechien, Mario Lentini, Pasquale Capaccio, Alberto Maria Saibene, Michele Gaffuri, Antonio Mario Bulfamante, Luca Giovanni Locatello, Portelli Tancredi, Ingrassia Angelo, Federico Sireci, Antonino Maniaci

**Affiliations:** ^1^ Department of Biomedical, Surgical and Dental Sciences University of Milan Milan Italy; ^2^ Division of Laryngology and Bronchoesophagology, Department of Otolaryngology Head Neck Surgery, EpiCURA Hospital, UMONS Research Institute for Health Sciences and Technology, Faculty of Medicine University of Mons (UMons) Mons Belgium; ^3^ ASP Ragusa Hospital Giovanni Paolo II Ragusa Italy; ^4^ Otolaryngology Unit, Santi Paolo e Carlo Hospital, Department of Health Sciences Università degli Studi di Milano Milan Italy; ^5^ Department of Clinical Sciences and Community Health University of Milan Milan Italy; ^6^ Pediatric Otolaryngology Unit, ASST Fatebenefratelli‐Sacco Buzzi Children Hospital Milan Italy; ^7^ Department of Otorhinolaryngology, University Hospital “Santa Maria Della Misericordia” Azienda Sanitaria Universitaria Friuli Centrale (ASUFC) Udine Italy; ^8^ Otorhinolaryngology Section, Biomedicine, Neuroscience and Advanced Diagnostic Department University of Palermo Palermo Italy; ^9^ Faculty of Medicine and Surgery University of Enna “Kore” Enna Italy

**Keywords:** aesthetic outcomes, extracorporeal septoplasty, nasal obstruction, nasal septal deviation, septoplasty techniques

## Abstract

**Objective:**

Extracorporeal septoplasty (ECS) is a surgical technique used to address severe nasal septal deviations, especially in patients in whom in situ septoplasty (ISS) is insufficient. This systematic review assesses the efficacy, safety, and clinical outcomes of ECS techniques, including conventional and modified ECS methods.

**Data Sources:**

PRISMA‐compliant systematic search of PubMed, Scopus, Web of Science, and Embase.

**Methods:**

Studies on ECS techniques were included. Eligibility criteria were established using the PICOTS framework. Study quality was assessed using the Cochrane Risk of Bias 2 tool and the Newcastle‐Ottawa Scale. Functional and aesthetic improvements were primary outcomes, while complication rates represented secondary outcomes.

**Results:**

Twenty‐two studies (retrospective, prospective, and RCTs) met the inclusion criteria. ECS was associated with significant functional improvement, as assessed by nasal obstruction scores measured by NOSE scores, acoustic rhinometry, and rhinomanometry. Aesthetic results were also satisfactory, with a significant improvement in the nasofrontal angle, nasolabial angle, and the tip projection index, as well as good patients' satisfaction. Complications were rare, with few cases of septal perforation, graft resorption, or residual nasal obstructive symptoms. Evidence strength was limited considering the majority of included studies were retrospective, with inherent bias risks, small sample sizes, and inconsistent follow‐up durations.

**Conclusions:**

ECS may be a successful and safe method for severe septal deviation correction, providing functional and aesthetic results with a low complication rate. More high‐quality, multicenter RCTs with long‐term follow‐up will be required for a standardization of surgical protocols and outcome measures.

**Level of Evidence:**

2.

## Graphical abstract

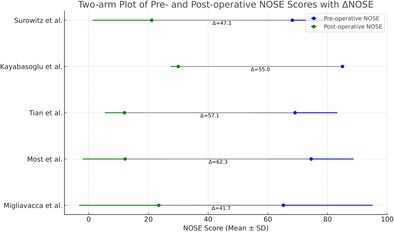

## 1 Introduction

Extracorporeal septoplasty (ECS) is a common surgical technique for the repair of extensive septal deformations in patients where intracorporeal or in situ septoplasty (ISS) does not achieve adequate structural adjustment and functional improvement [1, 2, 3]. This technique involves total excision of the septal cartilage, followed by reshaping and reinsertion, allowing for precise alignment and stabilization of the nasal framework [4, 5, 6]. ECS has been demonstrated to be especially beneficial in patients with severe caudal septal deviations, post‐traumatic deformities, and congenital abnormalities where previous methods may leave behind residual obstruction or deviation [7, 8].

In recent years, several changes in ECS techniques have been put forward to increase postoperative stability, limit complications, and improve both functional and aesthetic results. To improve long‐term results, innovation has focused on graft reinforcement techniques, suture fixation methods, and absorbable plates for providing structural support [9, 10, 11]. Modified ECS approaches have outperformed traditional ECS methods in this regard by decreasing the capacity of graft warpage, decreasing the incidence of postoperative septal deviation, and optimizing nasal air flow [12]. In addition, studies on the comparison of ECS to ISS suggest that ECS may provide better functional and aesthetic results in the long term, especially in cases where extensive septal correction is necessary [7, 12].

Even with these enhancements, graft resorption, septal perforation, and donor site morbidity are ongoing challenges of ECS, leading to continued investigation into the best modification and fixation techniques [8, 10]. Functional results after ECS are often assessed according to nasal obstruction symptom evaluation (NOSE) scores, acoustic rhinometry, or rhinomanometry, whereas aesthetic outcomes are determined through various anthropometric analyses and satisfaction surveys answered by the patient [8, 9, 11].

This systematic review is to evaluate the efficacy, safety, and comparative outcomes associated with ECS techniques, under both standard and modified methods. This review aims to provide current knowledge and insight regarding ECS technique and outcomes, presents the latest literature synthesized, investigates potentially optimal ECS modifications, and compares their efficacy to septoplasty alternatives, alongside complications, functional performance, and long‐term surgical efficacy.

## 2 Methods

This systematic review was reported in accordance with the Preferred Reporting Items for Systematic Reviews and Meta‐Analyses (PRISMA) guidelines [13] and was organized using the PICOTS framework (Population, Intervention, Comparator, Outcomes, Timing, and Study Design) to facilitate a complete and transparent assessment of the available ECS techniques. This protocol report was registered in Open Science Framework—OSF Public Registry, registry reference DOI https://doi.org/10.17605/OSF.IO/JC9TU (Center for Open Science, Charlottesville, VA, USA); the complete protocol is available at osf.io/jc9tu (accessed February 9, 2025).

### 2.1 Search Strategy

We performed a systematic literature search of the main medical databases—PubMed, Scopus, Web of Science, and Embase—to identify relevant studies. No limitation time was adopted to include all potentially relevant studies. Search terms included “extracorporeal septoplasty,” “ECS techniques,” “nasal septum deviation,” “functional septorhinoplasty,” and “aesthetic rhinoplasty.” Boolean operators (AND/OR) helped narrow the search. In particular, we applied the following search syntax (“Septoplasty” [MeSH] OR “Extracorporeal Septoplasty” [All Fields] OR “External Septoplasty” [All Fields] OR “Cartilage Graft Septoplasty” [All Fields] OR “Septal Reconstruction” [All Fields]) AND (“Nasal Obstruction” [MeSH] OR “Deviated Nasal Septum” [MeSH] OR “Nasal Septum” [MeSH] OR “Septal Deformity” [All Fields] OR “Nasal Deformity” [All Fields]) AND (“Treatment Outcome” [MeSH] OR “Surgical Procedures, Operative” [MeSH] OR “Postoperative Complications” [MeSH] OR “Recurrence” [MeSH] OR “Surgical Revision” [MeSH]).

Reference lists of included articles were also manually screened for additional relevant studies. The search was restricted to English language and to peer‐reviewed articles.

Inclusion and exclusion criteria were determined using the Population, Intervention, Comparator, Outcomes, Time Frame, Setting (PICOTS) framework [14]:

- Population (P): Patients who underwent extracorporeal septoplasty for severe nasal septal deviations (with functional and/or aesthetic complaints). Studies of pediatric populations or subjects with craniofacial syndromes were excluded.
- Intervention (I): ECS methods, namely conventional and modified approaches [4, 5, 6, 9].
- Comparator (C): Cohorts comparing ECS with in situ septoplasty (ISS) were accepted [12], but cohorts that evaluated ECS with no control group were also considered if they reported preoperative versus postoperative endpoints.
- Outcomes (O): the main outcome was functional improvement (e.g., compare anthropometric measures, nasal obstruction symptoms, NOSE score, and acoustic rhinometry), and aesthetic outcomes (e.g., compare anthropometric measures like nasolabial angle, nasofrontal angle, and tip projection index) [8, 9]. The secondary outcomes were surgery complications (such as graft resorption, perforation, infection) [10].
- Time (T): Studies with a postoperative follow‐up of < 3 months were excluded. Where available, long‐term follow‐up data were prioritized.
- Study Design (S): Any study design was considered eligible except for case reports, reviews, meta‐analyses, and editorials that were excluded from the present work.

### 2.2 Study Selection and Data Extraction

We observed the absence of uniform criteria for severe deviation across studies, with each author using its own clinical or radiologic thresholds for ECS eligibility. In addition, there was no consistency in reporting surgeon experience, ECS technique, follow‐up time, or outcomes.

Titles and abstracts were screened for relevance to the eligibility criteria by two independent review authors (M.L. and A.M.). In order to maximize inclusion, we assessed all articles that were deemed eligible by at least one reviewer for full‐text evaluation. Full‐text articles were obtained and independently assessed by the same reviewers, with disagreements resolved by a third reviewer (P.C.). A standardized extraction form was used to extract data on study design, sample size, demographics, ECS technique, outcomes, complications, and follow‐up duration.

### 2.3 Risk of Bias and Quality Assessment

We assessed the risk of bias using the Cochrane Risk of Bias 2 (RoB 2) tool for randomized controlled trials (RCTs) [15] and the Newcastle‐Ottawa Scale (NOS) for observational studies [16]. These tools enabled us to create a comprehensive assessment of several key bias domains.

The GRADE system [17] was reviewed to assess the overall quality of studies.

### 2.4 Data Synthesis and Statistical Analysis

A qualitative synthesis was conducted summarizing functional, aesthetic, and safety outcomes associated with reported ECS techniques. Due to heterogeneity in surgical techniques, outcome measures, and study designs, a formal meta‐analysis was not planned a priori or performed a posteriori. Rather, it provided a descriptive synthesis of data, with written emphasis on trends in functional improvement, aesthetic benefit, and complication/recurrence rates between ECS variations.

## 3 Results

### 3.1 Study Characteristics

**FIGURE 1:**
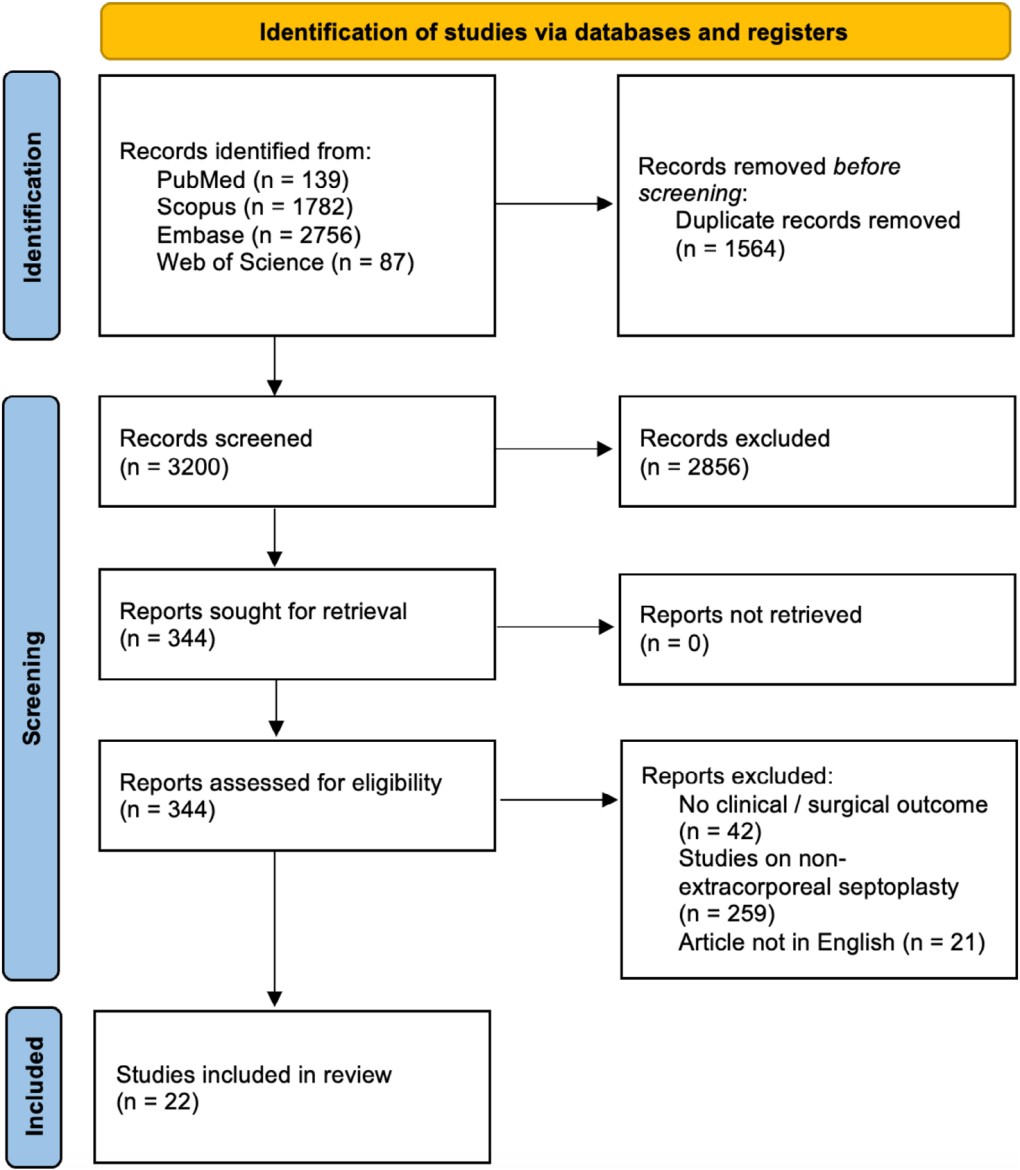
PRISMA flowchart for the study selection process of the present systematic review.

This systematic review included 22 studies published between 2005 and 2024, including 3761 patients, with study sample sizes ranging from 7 to 2119 patients (Figure 1). The studies were conducted in 13 different countries: the USA, Turkey, Italy, China, South Korea, Germany, Brazil, India, UK, Spain, Greece, Iran, and Switzerland, reflecting a geographically heterogeneous assessment of ECS techniques (Table 1). Gubisch et al. reported the largest study cohort in 2005 with 2119 patients, while the smallest sample size was found in the work of Most [18] with 23 patients. The study designs of the included studies varied, including retrospective [4, 5, 6, 8, 10, 11, 18, 19, 20, 21, 22, 23, 24, 25, 26, 27, 28, 29], prospective [9, 30, 31] and RCTs [7, 12]. Duration of follow‐up varied from 6 months to 6 years.

**TABLE 1:** Table reporting ECS techniques, study design, techniques assessed, and key outcomes.

Study	Sample size	Year of study	Geographical context	Study design	Techniques assessed	Additional procedures	Outcome measures	Follow‐up duration	Primary outcome tools	Complications reported	Strengths	Limitations	Grade assessment
Surowitz et al.	77 patients	2014	USA	Retrospective	Modified ECS	—	Long‐term nasal obstruction improvement, strong statistical analysis	12 months	NOSE score, VAS obstruction, Acoustic Rhinometry	Low complications, minor asymmetry	Large sample size, strong statistical methodology	Retrospective design, selection bias	Moderate
Kayabasoglu et al.	78 patients	2015	Turkey	Retrospective	Modified ECS	—	Novel modification for better nasal stability, reduced revision rates	Not specified	NOSE score, Patient‐reported outcomes	Not specified	Introduced new ECS modification	Limited sample reporting	Low
Loyo et al.	71 patients	2018	USA	Retrospective	Modified ECS	TR, FESS in 8 patients	ECS modification improved nasal stability and airflow	6 months	Rhinomanometry, CT scan analysis	Some postoperative edema	Moderate sample size, specific modification evaluation	Adjunct procedures may have influenced outcomes	Low
Gode et al.	40 patients (20 ECS, 20 ISS)	2018	Turkey	RCT	ECS vs. ISS	—	ECS vs. ISS: ECS superior in function & aesthetics	12 months	Acoustic Rhinometry, Patient satisfaction, VAS obstruction	Graft instability in some ISS cases	RCT design strengthens evidence for ECS vs. ISS	Small sample size, short follow‐up	High
Marangi et al.	50 patients	2018	Italy	Prospective	Modified ECS	—	Long‐term NOSE and AAR score improvements	24 months	Anthropometry, Nasal airway resistance	Minimal graft warping	Same surgeon performed procedures, reducing variability	Lack of randomization, limited recurrence data	Moderate
Vatamanesku et al.	7 patients	2020	Romania	Case Series	ECS	Unilateral TR in 1 patient	Detailed ECS technique documentation	Not specified	Descriptive surgical outcomes	Not reported	Detailed ECS technical guide for future studies	Extremely small sample size, study design	Very Low
Tian et al.	46 patients	2021	China	RCT	ECS	TR, FESS in 5 patients	RCT with extended follow‐up, functional and aesthetic gains	24 months	NOSE, Anthropometric analysis	Few cases of residual obstruction	Long‐term follow‐up, randomized design	Limited to a single country	High
Demir et al.	55 patients	2021	Turkey	Prospective	ECS	—	Prospective study, low complications, strong mixed‐method analysis	12 months	Nasal airflow dynamics, Patient satisfaction	Low rates of perforation	Low complication rates, mixed‐method analysis	Limited follow‐up period, reliance on anthropometry	Moderate
Mun et al.	64 patients	2021	South Korea	Retrospective	ECS	—	Anthropometric evaluation, patient satisfaction metrics	12 months	Nasal angles, Tip projection, Nasolabial angles	Some residual deviation reported	Anthropometric data adds objectivity to results	Retrospective design, possible selection bias	Low
Lee et al.	72 patients	2014	South Korea	Retrospective comparative	ECS vs. in situ septoplasty	—	Functional and aesthetic outcomes	12 months	Acoustic Rhinometry, VAS score volume	Dorsal irregularities	Direct comparison of techniques	Retrospective design	Moderate
Most et al.	12 patients	2006	USA	Prospective case series	Modified ECS with anterior reconstruction	Unilateral of Bilateral TR	Nasal airway function	6–12 months	NOSE scale	Minor complications only	Novel technique introduction	Small sample size	Low
Wilson et al.	46 patients	2011	USA	Retrospective case series	Modified ECS with simplified fixation	—	Complications and technical outcomes	Not specified	VAS obstruction	No major complications	Detailed technical description	Lack of standardized outcomes	Very low
Tasca et al.	133 patients	2018	Italy	Retrospective case series	ECS with valve stabilization	—	Nasal patency and satisfaction	12 months	Rhinomanometry and acoustic rhinometry	Minimal complications	Focus on functional outcomes	Retrospective design	Low
Rezaeian et al.	110 patients	2016	Switzerland	Prospective case series	ECS with keystone suturing	—	Stability of results	12 months	Not specified	No major complications	Novel suturing technique	Limited applicability	Low
Gubisch et al.	2119 patients	2005	Germany	Retrospective case series	Standard ECS	—	Functional and aesthetic outcomes	Not specified	Not specified	Minor complications in 5%–7%	Very large sample size	Lack of standardized outcomes	Low
Migliavacca et al.	27 patients	2024	Brazil	Prospective cohort	Modified ECS	—	Quality of life outcomes	16.8 months (mean)	NOSE and ROE scales	Minor complications	Patient‐centered outcomes	Small sample size	Moderate
Pradhan et al.	30 patients	2024	India	Retrospective cohort	Modified ECS	—	Functional and aesthetic outcomes	6 months	NOSE scale	Minor complications in 6.8%	Focus on crooked noses	Retrospective design	Low
Hacker et al.	245 patients	2021	UK	Retrospective comparative	Various rhinoplasty techniques	Bilateral Fracturing of turbinate	Functional and aesthetic outcomes	12 months	Revision Rate, Irregularities type at follow‐up	Not specified	Comparative design	Retrospective nature	Moderate
Serna et al.	26 patients	2014	Spain	Retrospective case series	Modified ECS	—	Functional and aesthetic outcomes	12 months	Not specified	Minor complications	Novel technique description	Small sample size	Low
Kantas et al.	64 patients	2008	Greece	Retrospective case series	ECS for crooked nose	—	Functional and aesthetic outcomes	12–36 months	Not specified	Minor complications	Focus on specific deformity	Lack of standardized outcomes	Low
Jang et al.	27 patients	2010	South Korea	Retrospective comparative	ECS vs. conventional septoplasty	—	Functional outcomes	12 months	VAS obstruction, Acoustic rhinometry	Minor complications	Large sample size	Retrospective design	Moderate
Sazgar et al.	23 patients	2014	Iran	Retrospective case series	ECS	—	Aesthetic outcomes	12 months	Photographic analysis	Minor complications	Focus on aesthetics	Lack of functional outcomes	Low

*Note:* The GRADE system was used to assess the quality of evidence: High (strong evidence from RCTs with low risk of bias), Moderate (prospective studies or large retrospective studies with some limitations), Low (retrospective studies with small sample sizes or short follow‐ups), and Very Low (case series or studies with high risk of bias and minimal statistical rigor).

Abbreviations: AAR, acoustic rhinometry assessment; ECS, extracorporeal septoplasty; ISS, in situ septoplasty; NOSE, nasal obstruction symptom evaluation; RCT, randomized controlled trial.

### 3.2 Functional Outcomes

We observed substantial heterogeneity across studies included regarding the severe septal deviation definition, ECS technique variations, surgeon experience levels, types, and timing of outcome parameters. Pooled analysis for 5/22 studies (*n* = 240 patients) reported NOSE outcomes changing from 72.2 ± 17.6 to 20.0 ± 15.9 postoperatively. Instead, 4/22 studies, including 190 patients, reported VAS obstruction reduction from 4.8 ± 2.5 to 3.2 ± 2.0. Average follow‐up period ranged from 6 months to 6 years, and most of the articles considered both functional (NOSE, VAS, acoustic rhinometry, etc.) and aesthetic (VAS) results. 5/22 studies reported additional procedures performed alongside ECS, including turbinate reduction [6, 7, 11, 18, 25], functional endoscopic sinus surgery [6, 7], and bilateral turbinates fracturing [25]. The majority of ECS was carried out with autogenous septal cartilage.

### 3.3 ECS Techniques

The studies encompassed a broad range of extracorporeal septoplasty (ECS) techniques, which were divided into modified ECS, traditional ECS, and comparative ECS approaches. Most of the studies centered on modifications designed to improve structural stability, functional outcomes, and decrease complications. Many modifications were made for better fixation techniques, strengthening of cartilage grafts, and improvement of long‐term nasal patency.

Surowitz et al. [4] instituted a reinforcement technique showing statistically significant long‐term improvement in nasal obstruction, and Kayabasoglu et al. [5] presented a new adjustment that intended to improve stability and reduce repeat surgeries. Loyo et al. [6] advanced grafting techniques for aesthetic and functional improvement. Marangi et al. [9] incorporated NOSE scores with acoustic rhinometry to validate the postoperative differences in a combined subjective and objective assessment.

Innovative ECS approaches were explored in several studies. Persichetti [29] provided important long‐term functional and structural outcomes data at 6 years' follow‐up. Rezaeian et al. [23] reported a keystone suturing technique to increase the stability of septal cartilage reconstruction. Most [18] and Wilson and Mobley [20] explored modifications for anterior reconstruction and simplified fixation, respectively.

In contrast, additional studies evaluated conventional ECS techniques, yielding useful preliminary data for contrasting outcomes between traditional and modified ECS methods. Gubisch [21] performed a large retrospective review of 2119 patients, providing an overview of standard ECS complications and outcomes. Demir et al. [10] prospectively studied 55 patients, noting the safety profile of traditional ECS with low complication rates. Mun et al. [8] provided a thorough assessment of aesthetic outcomes, including objective measurements like nasofrontal angle, nasolabial angle, and tip projection index.

Comparative studies were vital to assess how ECS stacked up against other methods. Gode et al. [12], Lee and Jang [19], and Jang and Kwon [27] compared ECS directly with in situ or conventional septoplasty and generally concluded that ECS is superior concerning both functional and aesthetic outcomes. Hacker et al. [25] provided a broader comparison of different rhinoplasty techniques, including ECS.

Repeatedly, these studies focused on specific applications or patient groups. Tian et al. [7] performed an RCT with extended follow‐up, while Migliavacca et al. [30] and Pradhan et al. [31] investigated modified ECS approaches in different geographical areas. Serna and Tapia [26] and Kantas et al. [22] explored ECS applications for specific nasal deformities.

### 3.4 Functional Outcomes and Aesthetic Outcomes

The NOSE score was the most widely used metric of outcomes [4, 7, 8, 9, 10, 12, 30, 31]. This provided a standardized measure of patient‐reported nasal obstruction symptoms, allowing for comparisons between different studies. Also, many studies included objective assessments of airflow either using acoustic rhinometry or rhinomanometry [7, 8, 9, 10, 24, 27, 28]. These methods provided an objective nasal patency and resistance measurement to supplement the subjective NOSE scores. This combination of subjective as well as objective measures enabled a comprehensive examination of functional improvements after ECS. Most studies [4, 5, 6, 7, 8, 9, 12, 22, 25, 27, 30] assessed patient satisfaction with functional outcomes using various questionnaires. Our emphasis on patient‐reported outcomes emphasizes the patient's perspective in their assessment of surgical success. As for the aesthetic outcomes, all the studies included in this review evaluated post‐surgical results, indicating the twofold functional‐aesthetic objectives of ECS. However, most studies [7, 8, 9, 10, 12, 22, 25] obtained objective measures of nasal geometry changes by the application of anthropometric analyses including nasofrontal angle, nasolabial angle, tip projection index. These numerical evaluations provided a consistent method of assessing aesthetic outcomes. One other commonly applied approach to assess aesthetic outcomes was through subjective evaluation by surgeons [4, 5, 6, 8, 9, 12, 23, 26, 29]. This established approach made use of senior clinicians' experience and expertise in making subjective judgments on aesthetic improvement. In a considerable number of the relevant studies [4, 5, 6, 7, 8, 9, 12, 22], the assessment of aesthetic results was based on the patient's satisfaction‐media value was achieved by optimal results, indicating their great role in successful procedure performance. Some of the studies used more complex or specialized assessment methodologies. Persichetti [29] also performed a long‐term follow‐up that gives information about the stability of aesthetic results over time. Rezaeian et al. [23] considering the aesthetic aspect of their new keystone suturing technique, Gubisch [21] performed a large‐scale evaluation of aesthetic outcomes in a sizable patient cohort. Comparative studies (e.g., Gode et al. [12] and Lee and Jang [19]) were especially helpful in contextualizing the aesthetic results of ECS for other septoplasty techniques. Overall, they concluded that ECS was superior in terms of achieving desirable aesthetic results, especially in cases of severe septal deviation.

### 3.5 Comparative Effectiveness and Safety Profile

Gode et al. [12] conducted a randomized controlled trial (RCT) comparing ECS to in situ septoplasty (ISS) directly, providing high‐level evidence in support of the benefits of ECS. They showed better functional and aesthetic results in ECS, with better nasal obstruction relief and better postoperative nasal symmetry than ISS. ECS was therefore strongly supported in this study, particularly in cases of severe septal deviations where traditional in situ techniques may be inadequate. Lee and Jang [19] and Jang and Kwon [27] also performed comparative studies confirming the results from Gode et al. [12]. The studies on ECS consistently demonstrated superior results over standard techniques of septoplasty, particularly for complex, obstructive deformities. Hacker et al. provided a comprehensive comparison of all types of rhinoplasties as well as ECS [25], placing ECS in the context of other rhinoplasty techniques. The safety of ECS was extensively studied in different studies. Gubisch [21] with its 2119 patient cohort, however, the authors showed very few (5%–7%) minor complications, giving strong and relevant information on ECS safety. Demir et al. [10] performed a prospective study of 55 patients and highlighted low complication rates with ECS. Persichetti [29] and Rezaeian et al. [23] also reported positive safety findings, with low rates of adverse events and the absence of significant septal perforation and graft resorption, and persistent nasal obstruction. The complication rates varied by surgical approach (2.9%–7.3%); Gubisch [21]. Many reported complications were minor and treatable, ranging from transient edema to mild asymmetries. Migliavacca et al. [30] demonstrated high patient satisfaction and low rates of post‐operative complaints and indirectly supported the safety profile of ECS by focusing on quality‐of‐life outcomes. To overcome this point, long‐term studies with extended follow‐up periods have addressed the long‐term safety and stability of ECS. Persichetti [29] with 6‐year follow‐up and Tian et al. [7], with a 2‐year follow‐up, both showed sustained good outcomes and few long‐term complications. These results suggest that the effects of ECS are durable and that the procedure does not carry new substantial long‐term risks. Notably, experimental studies involving modified ECS techniques, like Surowitz et al. [4], Kayabasoglu et al. [5], and Loyo et al. [6], also had a favorable safety profile for their novel approach. This indicates that even with the progress and refinement of ECS techniques, they are still performed with a great degree of safety.

### 3.6 Pooled Outcomes and Quantitative Analysis

**FIGURE 2:**
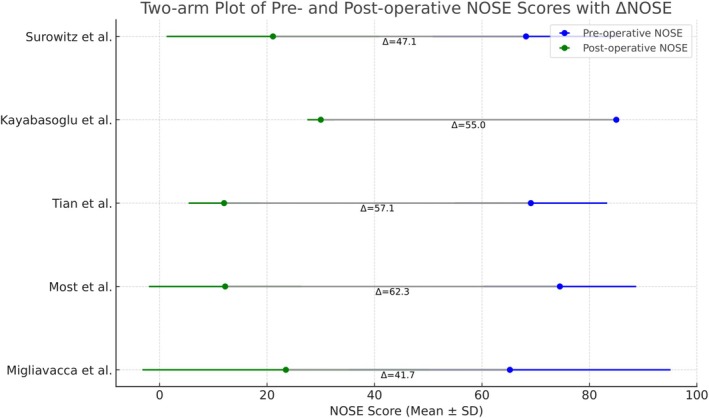
Pooled outcomes of NOSE scores at follow‐up.

We performed a pooled analysis on 5 studies [4, 5, 7, 18, 30], including a total of 240 patients to assess the effect of surgical intervention on NOSE scores. The NOSE score was significantly decreased at follow‐up based on the meta‐analysis fixed‐effects model, with a pooled mean difference of 49.6 (95% confidence interval (CI), 44.6–54.6; *Z* = 23.4; *p* < 0.001) (Figure 2). Substantial heterogeneity among the studies was found (*Q* = 167.32, df = 4, *p* < 0.001; *I* ^2^ = 97.6%), which is suggestive of high dissimilarity.

**FIGURE 3:**
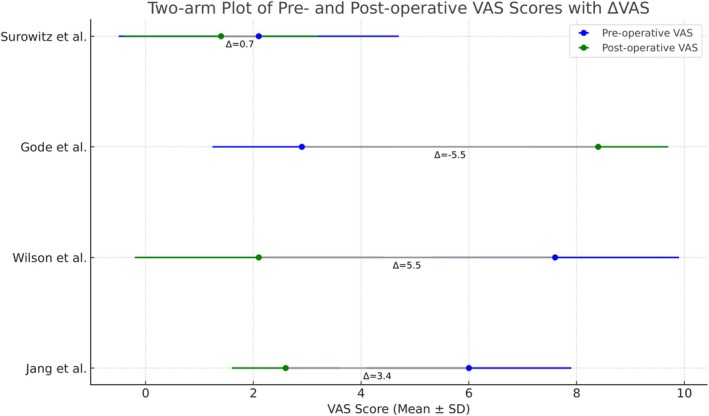
Pooled outcomes of VAS obstruction scores at follow‐up.

For VAS obstruction scores, we performed a pooled analysis of 4 studies [4, 12, 20, 27] comprising 190 patients. No significant difference was observed in the VAS obstruction scores between pre‐ and postoperative evaluations with a pooled mean difference of −0.01 (95% CI, −0.39 to 0.37; *Z* = −0.05; *p* = 0.958) according to the fixed‐effects model (Figure 3). Importantly, heterogeneity between studies was very high (*Q* = 475.18, df = 3, *p* < 0.001; *I* ^2^ = 99.4%), indicating large variability in study design and characteristics of patients.

### 3.7 Risk of Bias Assessment

We acknowledged an increased risk of selection and information bias inherent in the studies included in our dataset, given the predominance of retrospective studies, which limits the overall evidence level. Compared to retrospective studies, RCTs were less prone to selection bias, because patients were subjected to randomization, as can be appreciated in Supporting Information; however, retrospective studies are by nature at higher risk for selection bias. Blinding was also problematic among most surgical studies, as it was not possible owing to the nature of interventional studies; hence, performance bias was difficult to prevent. Detection bias was variable: studies using objective outcome measures such as NOSE scores, rhinomanometry, and acoustic rhinometry had a lower risk of this bias than those dependent upon subjective measures. Follow‐up duration and participant retention were reviewed to evaluate attrition bias; longer follow‐up periods resulted in a lower risk of bias. We examined reporting bias by reviewing outcomes for clarity and completeness. Quality of evidence was high for well‐conducted RCTs, moderate for well‐designed prospective studies, and low for retrospective studies with methodological limitations.

## 4 Discussion

This systematic review of 22 studies supports the efficacy of extracorporeal septoplasty (ECS) for the management of severe septal deviations. However, the quality of evidence was a major limitation, considering that most of the included studies (17/22) [4, 5, 6, 8, 21, 22, 26] were retrospective study designs. This methodological weakness introduces a higher risk of selection and information biases, thus reducing the strength and generalizability of our conclusions. In addition, the surgical indications for ECS remain an important point of debate.

We found no uniform criteria for ECS candidacy, with most studies including patients with severe caudal septal deviations, post‐traumatic deformities, and complex nasal anatomy.

Although some authors such as Surowitz et al. [4] and Kayabasoglu et al. [5] proposed specific indications for ECS in severe anterior deviation or failed prior septoplasty, this was not universally accepted.

The only randomized controlled trial of Gode et al. [12] was valuable but was limited in sample size and duration of follow‐up, limiting generalizability. Moreover, we found a lack of standardized definitions for “severe septal deviation” and inconsistent reporting of surgical expertise and ECS technical variations. This calls for high‐quality, prospective studies with larger cohorts and longer follow‐up periods to definitively determine the long‐term efficacy and safety profile of ECS.

Notably, a significant gap in the literature is the absence of standardized definition criteria for severe or complex septal deviations. Such absence limits patient selection protocols and adds complexity to cross‐study comparisons. While the studies by Surowitz et al. [4], Kayabasoglu et al. [5], and Loyo et al. [6] have sought to establish criteria for ECS candidacy, no standardized approach exists. A validated grading system for septal deviations should be developed, as is done for other nasal pathologies, to standardize the definition of septal deviation within studies and enable comparison between them.

The value of preoperative imaging—particularly computed tomography (CT) scans—to guide ECS is controversial. Although CT images can yield significant information on bony deviations, it is not widely accepted for routine use. Studies like Demir et al. [10] have delved into the utility of CT scans in septoplasty assessment, but the precise role of CT in ECS planning needs further exploration. Future studies are warranted to evaluate how preoperative CT scans affect surgical planning and outcomes, which can help establish evidence‐based guidelines for candidate treatment with ECS.

Surgeon experience is an often‐overlooked variable in ECS outcomes. The complexity of the technique requires advanced skills in septoplasty and rhinoplasty, but few studies have adequately assessed the learning curve of ECS. This variability in surgical expertise may substantially influence the reported outcomes. Studies like Wilson and Mobley [20] have addressed this problem, but a more complete analysis is necessary. Further studies in this area should stratify outcomes by surgeon experience to describe the efficacy and safety of ECS.

More studies are needed to compare the effectiveness of ECS versus in situ septoplasty (ISS). While studies such as Gode et al. [12], Lee and Jang [19], and Jang and Kwon [27] suggest the relative superiority of ECS, especially in complex cases, the possibility of selection bias in non‐randomized comparisons cannot be discounted. In addition, as reported by Surowitz et al. [4] and Wilson and Mobley [20], the increased surgical complexity and operative time of ECS over ISS is worth questioning in terms of cost‐effectiveness and appropriateness for less severe deviations.

Lastly, although reported complication rates are low overall [10, 21], the risk for long‐term complications, including warping or resorption of the graft, calls for prolonged follow‐up periods in future studies. An additional debated topic is the graft material used for ECS reconstruction. While most studies utilized septal cartilage, some described supplementary grafting with costal or auricular cartilage in cases of insufficient septal tissue. However, specific subgroup analysis on such graft resorption or warping was not reported, resulting in missing comparative studies based on graft type.

While Persichetti [29] offered great long‐term data, additional long follow‐up studies are required.

### 4.1 Literature Limitations

Although this systematic review has shown positive outcomes following ECS, studies of low quality were included. Most of the included studies were retrospective designs, which are inherently prone to selection, information, and reporting biases. This weakens the level of evidence and highlights the need for well‐designed prospective and randomized controlled trials to validate the reported outcomes. Moreover, we found a lack of clear and uniform criteria to define advanced or complex septal deviations, with variable thresholds applied clinically or radiologically. While validated grading systems exist for other nasal pathologies such as the nasal polyp scoring (NPS) or the International Frontal Activity Classification (IFAC) for the frontal sinus [4, 5], there is currently no agreed‐upon classification system that aids in determining ECS indications. Such heterogeneity complicates comparisons between studies and highlights the need for a grading system to characterize severe septal deviations. Again, the patient selection criteria for ECS are fraught with controversy. Preoperative assessment with nasal endoscopy should be sufficient, and the role of computed tomography is still an open question. The value of preoperative imaging, as CT scans for ECS planning, remains debated. Although CT imaging may provide detailed anatomical bony information, it is not routinely used in clinical practice. Lastly, another often neglected factor that has a great impact on the ECS outcome is the surgeon's experience with the surgical technique. ECS is a much more complex procedure than classic septoplasty, involving significant technical expertise in both septoplasty and rhinoplasty. Few studies account for the learning curve that may be driving outcome variation. ECS variations, techniques, training received, case volume, and proficiency may contribute to the heterogeneity in outcomes observed across studies. Future series should be stratified so that a more accurate assessment of the real utility and safety of ECS can be obtained.

## 5 Conclusion

ECS is still an effective technique for correcting septal deviations with great improvements in both functional and aesthetic outcomes. Compared with ECS or ISS, modified ECS methods have exhibited better stability and higher patient satisfaction. Although complication rates are low thus far, the long‐term force stability of grafts, as well as revision rates, warrant further study. Future studies should emphasize high‐quality, prospective research reporting standardized definitions of severe deviation, specific indications for the technique, surgeon experience, and detailed ECS techniques. In addition, the clinical utility and cost‐effectiveness of preoperative imaging, including CT scans, follow‐up durations on long‐term outcomes, are easier to compare, including resorption and warping.

## Conflicts of Interest

The authors declare no conflicts of interest.

## Supporting information


**Data S1:** Risk of bias assessment.

## Data Availability

The data that support the findings of this study are available from the corresponding author upon reasonable request.
