# Supplementary material for: Extracorporeal Septoplasty for Severe Nasal Septal Deviation: A Systematic Review
Source: Laryngoscope Investig Otolaryngol. 2025 Oct 10;10(5):e70280. doi: 10.1002/lio2.70280 (PMC12512901; doi:10.1002/lio2.70280)
Supplement: Supplementary file 1 — Data S1: Risk of bias assessment. [file LIO2-10-e70280-s001.docx]

| **Author et al.** | **Cochrane Risk of Bias 2 (RoB 2) for RCTs** | **Newcastle-Ottawa Scale (NOS) for Observational Studies** |
| --- | --- | --- |
| Surowitz et al. | Not applicable | ★★★★★★★☆☆ (7/9)<br>Selection: ★★★★Comparability: ★★<br>Outcome: ★☆ (12-month follow-up but possible selection bias) |
| Kayabasoglu et al. | Not applicable | ★★★★★☆☆☆☆ (5/9) Selection: ★★★☆ Comparability: ★★ Outcome: ☆☆ (Follow-up duration not specified) |
| Loyo et al. | Not applicable | ★★★★★★☆☆☆ (6/9)<br>Selection: ★★★☆<br>Comparability: ★★<br>Outcome: ★☆ (Only 6-month follow-up) |
| Gode et al. | Moderate Risk of Bias<br>Randomization: Low risk<br>Allocation concealment: Some concerns<br>Blinding participants: High risk (surgical)<br>Blinding assessors: Some concerns<br>Missing data: Low risk<br>Selective reporting: Low risk<br>Other bias: Some concerns (small sample of 40 patients) | Not applicable |
| Marangi et al. | Not applicable | ★★★★★★★☆☆ (7/9)<br>Selection: ★★★☆<br>Comparability: ★★<br>Outcome: ★★ (24-month follow-up) |
| Vatamanesku et al. | Not applicable | ★★★☆☆☆☆☆☆ (3/9)<br>Selection: ★★☆☆<br>Comparability: ★☆<br>Outcome: ☆☆ (Extremely small sample size of 7 patients) |
| Tian et al. | Some Concerns<br>Randomization: Low risk<br>Allocation concealment: Some concerns<br>Blinding participants: High risk (surgical)<br>Blinding assessors: Low risk<br>Missing data: Low risk<br>Selective reporting: Low risk<br>Other bias: Low risk (24-month follow-up) | Not applicable |
| Demir et al. | Not applicable | ★★★★★★★☆☆ (7/9)<br>Selection: ★★★★<br>Comparability: ★★<br>Outcome: ★☆ (12-month follow-up) |
| Mun et al. | Not applicable | ★★★★★★☆☆☆ (6/9)<br>Selection: ★★★☆<br>Comparability: ★★<br>Outcome: ★☆ (Retrospective design) |
| Lee et al. | Not applicable | ★★★★★★★☆☆ (7/9)<br>Selection: ★★★★<br>Comparability: ★★<br>Outcome: ★☆ (Comparative design strengthens study) |
| Most | Not applicable | ★★★★★☆☆☆☆ (5/9)<br>Selection: ★★★☆<br>Comparability: ★☆<br>Outcome: ★☆ (Small sample size of 23 patients) |
| Wilson et al. | Not applicable | ★★★★☆☆☆☆☆ (4/9)<br>Selection: ★★☆☆<br>Comparability: ★☆<br>Outcome: ★☆ (Lack of standardized outcomes) |
| Tasca et al. | Not applicable | ★★★★★★☆☆☆ (6/9)<br>Selection: ★★★☆<br>Comparability: ★★<br>Outcome: ★☆ (Large sample of 133 patients) |
| Rezaeian et al. | Not applicable | ★★★★★★☆☆☆ (6/9)<br>Selection: ★★★☆<br>Comparability: ★★<br>Outcome: ★☆ (Novel technique but limited applicability) |
| Gubisch et al. | Not applicable | ★★★★★★☆☆☆ (6/9)<br>Selection: ★★★★<br>Comparability: ★☆<br>Outcome: ★☆ (Very large sample of 2119 patients but lack of standardized outcomes) |
| Migliavacca et al. | Not applicable | ★★★★★★★☆☆ (7/9)<br>Selection: ★★★★<br>Comparability: ★★<br>Outcome: ★☆ (Mean follow-up of 16.8 months) |
| Pradhan et al. | Not applicable | ★★★★★★☆☆☆ (6/9)<br>Selection: ★★★☆<br>Comparability: ★★<br>Outcome: ★☆ (Short 6-month follow-up) |
| Hacker et al. | Not applicable | ★★★★★★★☆☆ (7/9)<br>Selection: ★★★★<br>Comparability: ★★<br>Outcome: ★☆ (Large sample of 182 patients) |
| Serna et al. | Not applicable | ★★★★★☆☆☆☆ (5/9)<br>Selection: ★★★☆<br>Comparability: ★☆<br>Outcome: ★☆ (Small sample size of 26 patients) |
| Kantas et al. | Not applicable | ★★★★★★☆☆☆ (6/9)<br>Selection: ★★★☆<br>Comparability: ★★<br>Outcome: ★☆ (Long follow-up of 12-36 months) |
| Jang et al. | Not applicable | ★★★★★★★☆☆ (7/9)<br>Selection: ★★★★<br>Comparability: ★★<br>Outcome: ★☆ (Large sample of 100 patients) |
| Sazgar et al. | Not applicable | ★★★★★☆☆☆☆ (5/9)<br>Selection: ★★★☆<br>Comparability: ★☆<br>Outcome: ★☆ (Focus only on aesthetic outcomes) |

Results from the risk of bias assessment risk of bias using the Cochrane Risk of Bias 2 (RoB 2) tool for randomized controlled trials (RCTs) and the Newcastle-Ottawa Scale (NOS) for observational studies.
